# Validation of the bag‐mediated filtration system for environmental surveillance of poliovirus in Nairobi, Kenya

**DOI:** 10.1111/jam.14807

**Published:** 2020-08-14

**Authors:** C.S. Fagnant‐Sperati, Y. Ren, N.A. Zhou, E. Komen, B. Mwangi, J. Hassan, A. Chepkurui, R. Nzunza, J. Nyangao, W.B. van Zyl, M. Wolfaardt, P.N. Matsapola, F.B. Ngwana, S. Jeffries‐Miles, A. Coulliette‐Salmond, S. Peñaranda, E. Vega, J.H. Shirai, A.L. Kossik, N.K. Beck, D.S. Boyle, C.C. Burns, M.B. Taylor, P. Borus, J.S. Meschke

**Affiliations:** ^1^ Department of Environmental and Occupational Health Sciences University of Washington Seattle WA USA; ^2^ Department of Biostatistics University of Washington Seattle WA USA; ^3^ Centre for Viral Research Kenya Medical Research Institute Nairobi Kenya; ^4^ Department of Medical Virology University of Pretoria Pretoria South Africa; ^5^ Cherokee Nation Assurance a contracting agency to the Division of Viral Diseases Centers for Disease Control and Prevention Atlanta GA USA; ^6^ Division of Viral Diseases Centers for Disease Control and Prevention Atlanta GA USA; ^7^ PATH Seattle WA USA

**Keywords:** enteric viruses, environmental surveillance, filtration, Kenya, poliovirus, ViroCap, wastewater

## Abstract

**Aims:**

This study compared the bag‐mediated filtration system (BMFS) and standard WHO two‐phase separation methods for poliovirus (PV) environmental surveillance, examined factors impacting PV detection and monitored Sabin‐like (SL) PV type 2 presence with withdrawal of oral polio vaccine type 2 (OPV2) in April 2016.

**Methods and Results:**

Environmental samples were collected in Nairobi, Kenya (Sept 2015–Feb 2017), concentrated via BMFS and two‐phase separation methods, then assayed using the WHO PV isolation algorithm and intratypic differentiation diagnostic screening kit. SL1, SL2 and SL3 were detected at higher rates in BMFS than two‐phase samples (*P* < 0·05). In BMFS samples, SL PV detection did not significantly differ with volume filtered, filtration time or filter shipment time (*P *> 0·05), while SL3 was detected less frequently with higher shipment temperatures (*P* = 0·027). SL2 was detected more frequently before OPV2 withdrawal in BMFS and two‐phase samples (*P* < 1 × 10^−5^).

**Conclusions:**

Poliovirus was detected at higher rates with the BMFS, a method that includes a secondary concentration step, than using the standard WHO two‐phase method. SL2 disappearance from the environment was commensurate with OPV2 withdrawal.

**Significance and Impact of the Study:**

The BMFS offers comparable or improved PV detection under the conditions in this study, relative to the two‐phase method.

## Introduction

Monitoring poliovirus (PV) circulation is critical to vaccination efforts and eradication certification. Only wild PV (WPV) type 1 remains, with 165 cases reported in 2019 (World Health Organization (WHO) 2019a); the last detection of WPV type 3 was in 2012 (Kew *et al*. 2014). WPV type 2 and WPV type 3 were declared eradicated in 2015 (Diop 2017) and 2019 (WHO 2019b) respectively. Eradication of WPV type 2 and the frequent emergence of type 2 circulating vaccine‐derived poliovirus (VDPV) prompted PV type 2 (PV2) withdrawal from the oral polio vaccine (OPV) in April 2016, changing the vaccine from trivalent (tOPV) to bivalent (bOPV) (WHO 2013; Maes *et al*. 2017; Tevi‐Benissan *et al*. 2017). While clinical surveillance for acute flaccid paralysis (AFP) is the Global Polio Eradication Initiative’s (GPEI) gold standard surveillance approach, environmental surveillance is an important supplement for detecting PV circulation in the absence of AFP cases (Hovi *et al*. 1986; Kopel *et al*. 2014; WHO 2015a; Duintjer Tebbens *et al*. 2017; Koopman *et al*. 2017).

Environmental surveillance for PV in Nairobi, Kenya began in October 2013 (Borus *et al*. 2015; WHO 2017). Kenya’s final clinical WPV case occurred in July 2013, and the last detected WPV environmental sample was in October 2013 (Centers for Disease Control and Prevention (CDC) 2013; Kamadjeu *et al*. 2014; Borus *et al*. 2015; WHO 2015a, 2017). In 2015, environmental surveillance expanded to Mombasa, Garissa and Kisumu (WHO 2017). Environmental surveillance in Kenya utilizes the standard WHO procedure (two‐phase method): a 500 ml grab sample is concentrated by two‐phase separation, for a 50‐fold concentration factor and 10 ml final volume (WHO 2015a). This method has been used for over 30 years; nevertheless, the GPEI recommended evaluation of alternative environmental surveillance methods (Pöyry *et al*. 1988; WHO 2015a, 2015b). Consequently, the bag‐mediated filtration system (BMFS) was developed to enable primary concentration of 3–6 l in the field, followed by secondary concentration in the laboratory. This method increased the concentration factor to 300‐ to 600‐fold with a final volume of 10 ml (Fagnant *et al*. 2014, 2018; WHO 2015a; Zhou *et al*. 2018). A previous study was conducted in Nairobi to identify and address complications from conducting a multi‐national study, and compare PV detection between environmental samples concentrated by the two‐phase method and BMFS, using a limited data set (Zhou *et al*. 2018).

The objectives of the study described here were to (i) validate the BMFS for PV environmental surveillance with the two‐phase method, (ii) examine sample processing factors that may impact PV detection and (iii) monitor environmental Sabin‐like PV type 2 (SL2) presence before and after the withdrawal of OPV2.

## Materials and methods

### Study design

From 29 September 2015 to 14 February 2017, samples were collected in Nairobi (*n* = 133) twice per month from four sites: Starehe, Eastleigh A, Eastleigh B and Kibera (described in Supporting Information). Single water samples were collected within 5 min and a 1‐m radius of each other for parallel testing by the BMFS and two‐phase concentration methods. Each collected BMFS water sample was concentrated using two ViroCap™ filters, resulting in two replicate BMFS samples for each BMFS sampling event.

**Figure 1:**
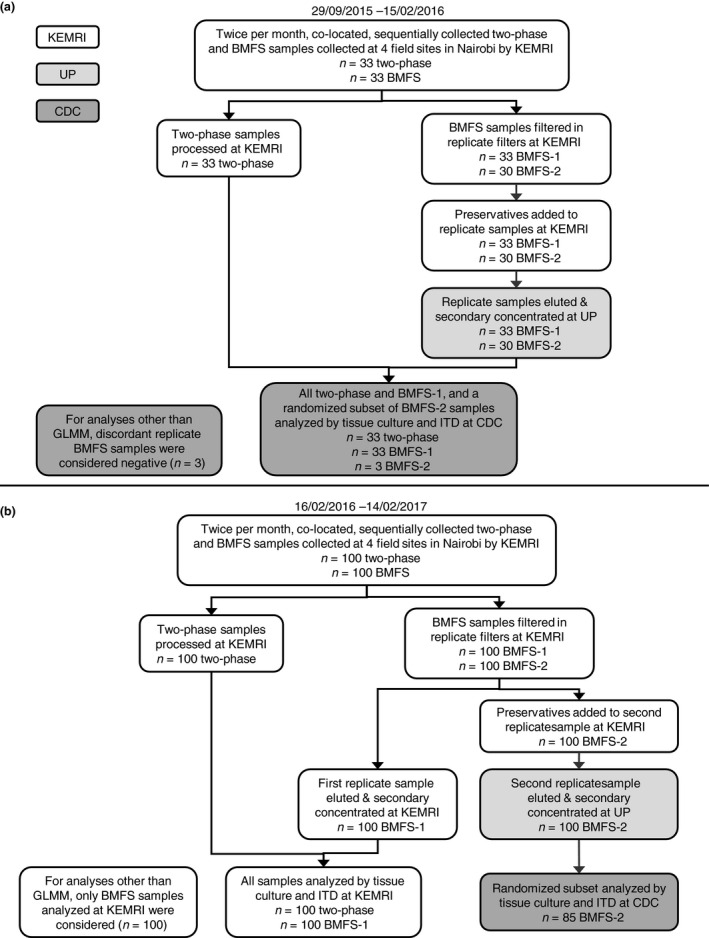
Kenya bag‐mediated filtration system (BMFS) and two‐phase separation method comparison study design. (a) Comparison of two‐phase and BMFS samples, 29 September 2015–15 February 2016. (b) Comparison of two‐phase and BMFS samples, 16 February 2016–14 February 2017. KEMRI is Kenya Medical Research Institute. UP is University of Pretoria. CDC is Centers for Disease Control and Prevention. BMFS is bag‐mediated filtration system. ITD is intratypic differentiation. GLMM is generalized linear mixed model.

Primary concentration for two‐phase and replicate BMFS samples occurred at Kenya Medical Research Institute (KEMRI) in Nairobi throughout the study. Additional processing and analyses occurred at multiple locations (KEMRI, University of Pretoria (UP), and/or CDC) during this study. From 29 September 2015 to 15 February 2016 (Fig. 1a), replicate BMFS filters were treated with preservatives at KEMRI, shipped to UP in Pretoria, South Africa, for processing, and then to CDC in Atlanta, United States, where a randomized portion of BMFS samples was analysed. After two‐phase separation was performed at KEMRI, all two‐phase sample concentrates were shipped to CDC for analysis. On 16 February 2016, KEMRI personnel were trained to fully process BMFS samples by the University of Washington personnel to perform virus isolation on environmental samples by WHO‐AFR personnel. From 16 February 2016 to 14 February 2017 (Fig. 1b), one BMFS filter was treated with preservatives at KEMRI, shipped to UP for processing and then to CDC where a randomized portion was analysed. The second BMFS filter received no preservative treatment and remained at KEMRI for processing and analysis. All BMFS samples remaining at KEMRI were analysed, and all two‐phase samples were processed and analysed at KEMRI.

### BMFS samples

Eight‐litre samples were collected in a collection bag, then sealed and placed into a water‐tight, insulated bucket, with cold packs, for transport to KEMRI within 4 h (i.e. bucket protocol) (Zhou *et al*. 2018), and filtration within 24 h. Collection bags were hung on a tripod stand outside on KEMRI’s campus, allowed to settle 15 min and approximately 0·5 l was drained as waste to remove settled solids. A Y‐adapter was connected to the bag’s outlet, two replicate ViroCap filters preseeded with a known titre of bacteriophage MS2 as previously described (Zhou *et al*. 2020) were attached to either end and samples were filtered simultaneously by gravity.

For filters shipped to UP, a 2% sodium benzoate (Becton Dickinson, Sparks, MD) and 0·2% calcium propionate (Becton Dickinson) preservative mixture was passed through the filter at KEMRI (Fagnant *et al*. 2017a). All filters were processed by a single 30‐min elution using 100 ml pH 9·5 eluent containing 1·5% beef extract (Becton Dickinson) and 0·05 mol l^−1^ glycine (Fisher Scientific, Hampton, VA (KEMRI); Merck KGaA, Darmstadt, Germany (UP)) (Fagnant *et al*. 2017b, 2018; Zhou *et al*. 2018). Secondary concentration was performed on the eluate by polyethylene glycol (PEG) precipitation (Meleg *et al*. 2008; Kiulia *et al*. 2010), with addition of 14 g PEG 8000 (Sigma Aldrich (KEMRI); Amresco LLC, Solon, OH (UP)) 1·17 g sodium chloride (NaCl) (Sigma Aldrich), overnight incubation (room temperature (KEMRI) or 4°C (UP)), and centrifugation (2500 ***g*** (KEMRI) or 6500 ***g*** (UP), 30 min). The pellet was resuspended in 10 ml PBS.

For BMFS samples processed at UP the following controls were included. Infectious MS2 preseeded onto the ViroCap filters as a BMFS process control, was enumerated in the filter eluate via the double agar layer method using an *E. coli* F^−^amp host as previously described (Adams 1959; US EPA 2000; Zhou *et al*. 2020). MS2 recovery efficiency ranged from 0 to 5900%, with a median of 9·9%. Additionally, an aliquot of the resuspended secondary concentration pellet remained at UP. These samples were chloroform extracted, seeded with 5 × 10^4^ copies of mengovirus as an extraction control and nucleic acid extracted via the semi‐automated NucliSENS® easyMAG® instrument (bioMérieux, SA, Marcy‐I’Étoile, France) (Zhou *et al*. 2020). The median extraction efficiency for mengovirus was 32·04% (interquartile range = 20·18–52·61%). Real‐time reverse transcription polymerase chain reaction (rRT‐PCR) analysis using CeeramTools® (bioMérieux) showed that 98·9% samples were positive for mengovirus. The RNA from samples that tested negative for mengovirus was diluted 10‐fold and all tests were repeated.

### Two‐phase samples

One‐litre samples were collected, placed in a cooler with ice packs, transported to KEMRI within 4 h, and concentrated by two‐phase separation within 48 h (WHO 2015a). A 500 ml aliquot was centrifuged to pellet debris and saved. The supernatant was combined with 287 ml 29% PEG 6000, 39·5 ml 22% dextran T40 (Pharmacosmos, Holbaek, Denmark) and 35 ml 5 mol l^−1^ NaCl, and placed into a separation funnel at 4°C overnight. The lower‐ and inter‐phases were collected, and the pellet was added to the concentrate. Secondary concentration was not performed, according to the standard WHO protocol (WHO 2015a).

### Assay

Concentrates were chloroform extracted and assayed at KEMRI or CDC via the WHO Poliovirus Isolation Algorithm, utilizing L20B (mouse L cell expressing the PV receptor, CD155) and human rhabdomyosarcoma (RD) cell lines (WHO 2015a). Samples positive for cytopathic effects (CPE) were screened by rRT‐PCR using a suite of assays included in the Poliovirus Intratypic Differentiation rRT‐PCR Kit (CDC, Atlanta, USA) on an Applied Biosystems® 7500 thermocycler (Applied Biosystems, Foster City, CA), as previously described (Gerloff *et al*. 2018). Briefly, reaction cycling conditions included: reverse transcription (RT) at 50°C for 30 min, RT inactivation and initial denaturation at 95°C for 1 min, followed by 40 cycles of 95°C for 15 s, 50°C for 45 s and 72°C for 5 s with a 25% ramp rate between the annealing and elongation step. Results were reported following the PV diagnostic algorithm (Kilpatrick *et al*. 2009; WHO 2015a; Gerloff *et al*. 2018). Briefly, samples positive for CPE in RD cells, but negative in L20B cells are reported as nonpolio enterovirus (NPEV). Additionally, samples positive for CPE, but negative for the following assays (pan enterovirus (PanEV), Sabin‐like PV type 1 (SL1), Sabin‐like PV type 2 (SL2), Sabin‐like PV type 3 (SL3), pan poliovirus (PanPV), WPV1, PV type 2, WPV3‐I and WPV3‐II) are reported as non‐enterovirus. If the PanEV assay is positive and others negative, samples are reported as NPEV. Samples presumptively positive for Sabin‐like PV are further assayed for VDPV type 1 or VDPV type 3, and sequenced if determined to be non‐Sabin‐like (NSL) or reported as Sabin‐like. Any NSL, PV2 positive or indeterminate samples are sequenced for final confirmation (Gerloff *et al*. 2018).

### Analyses

Statistical analyses were conducted using Microsoft® Excel 2016 (concentration factor, effective volume assayed, and Pearson’s chi‐squared) or RStudio® ver. 1.0.143 using the lme4, dplyr and Rcpp packages (McNemar mid‐*P*, generalized linear mixed model (GLMM) and logistic regression) (additional details on these methods are provided in the Statistical Methods section of the Supporting Information).

The concentration factor (ratio between the original and final sample volumes) and effective volume assayed (product of the concentration factor and assay volume) were calculated. The WHO algorithm assay volume is 3 ml.

The McNemar mid‐*P* test was used to determine significant differences between BMFS and two‐phase samples (Objective 1) (McNemar 1947; Fagerland *et al*. 2013; Zhou *et al*. 2018). As the location where two‐phase samples were analysed switched from the CDC to KEMRI on 15 February 2016, BMFS samples compared included those analysed at CDC prior to 15 February 2016 (single and/or replicate samples) and at KEMRI afterwards (single samples) to best match the BMFS and two‐phase sample analysis (Table S2), with the values used shown in the 2x2 tables in Table 1. Replicate BMFS samples were combined and considered positive for the target only if both replicates tested positive (*n* = 3) (Zhou *et al*. 2018). Note, BMFS and two‐phase samples were not processed from the same homogenous source, though are matched temporally (≤5 min) and spatially (≤1‐m radius). The odds ratio (OR) of virus detection in BMFS samples compared to virus detection in two‐phase samples and 95% confidence intervals (CI) was calculated (Zhou *et al*. 2018) (Microsoft Excel 2016).

Generalized linear mixed model and logistic regression models were performed to determine the effect of multiple variables on detection of SL1, SL2, SL3, NPEV and any PV (Tables 2 and 3 and Table S2). The effect of concentration method (BMFS or two‐phase) on PV detection was tested (Objective 1). Factors tested for their impact on PV detection in BMFS samples included (i) filtration volume and time (GLMM), (ii) processing time (CDC samples, GLMM; KEMRI samples, logistic regression), (iii) refrigerated shipping conditions (shipping time, GLMM; temperature, logistic regression) and (iv) assay location (GLMM) (Objective 2). Logistic regression was used to determine effect of assay location on PV detection for two‐phase samples (Objective 2). The effects of volume and time filtered on PV detection were not tested for two‐phase samples, as the processed volume did not vary (500 ml) and no filtration occurred. Processing time was not tested for two‐phase since all two‐phase samples were received the day of collection, and processed within 2 days (WHO 2015a). The effect of refrigerated shipping of BMFS filters on PV detection was tested for BMFS samples assayed at CDC (BMFS samples assayed at KEMRI were not shipped), but not for two‐phase and BMFS concentrates, as these were shipped frozen, with minimal temperature fluctuation.

**Table 1:** Comparison of PV detection in sequentially collected matched BMFS and two‐phase samples

SL1		Two‐phase		SL2		Two‐phase		SL3		Two‐phase	
		+	−			+	−			+	−
BMFS	+	14	19	BMFS	+	21	19	BMFS	+	32	39
	−	8	92		−	6	87		−	10	52

PV, poliovirus; SL1, Sabin‐like PV type 1; SL2, Sabin‐like PV type 2; SL3, Sabin‐like PV type 3; BMFS, bag‐mediated filtration system.

**Table 2:** Virus detection in sequentially collected matched BMFS and two‐phase samples

	SL1	SL2	SL3	NPEV	PV Negative	*n* (BMFS/Two‐phase)	
Comparison of selected BMFS* and two‐phase samples						
BMFS (%)	24·8	30·1	53·4	51·1	27·8	133*	
Two‐phase (%)	16·5	20·3	31·6	67·7	49·6	133	
OR (CI)	2·38 (1·04, 5·43)	3·17 (1·23, 7·93)	3·90 (1·95, 7·81)	0·41 (0·22, 0·74)	0·19 (0·09, 0·44)	133*/133	
McNemar mid‐*P*‐value	0·036	0·009	<0·001	0·002	<0·001	133^a^/133	
Comparison of all BMFS^†^ and two‐phase samples						
OR (CI)^‡^	2·79 (1·32, 5·87)	3·53 (1·40, 8·89)	3·57 (2·01, 6·32)	0·50 (0·29, 0·85)	0·28 (0·15, 0·50)	221^†^/133	
GLMM *P*‐value^‡^	0·007	0·007	<0·001	0·010	<0·001	221^†^/133	

GLMM, generalized linear mixed model; NPEV, nonpolio enterovirus; PV negative, no poliovirus detected in sample; OR, odds ratio; CI, 95% confidence interval boundaries.

* BMFS samples included single (*n* = 30) and combined (*n* = 3) replicate samples analysed at CDC (29 September 2015 through 15 February 2016) and single samples analysed at KEMRI (*n* = 100; 16 February 2016 through 14 February 2017).

^†^ All BMFS samples were included in the analysis.

^‡^ Adjustment factors for all analyses included sample site (categorical), season (binary) and pair (cluster). The SL2 analysis additionally included bOPV switch (binary). See Table S4 for a detailed explanation of the adjustment factors included.

The GLMM accounted for random and fixed effect variables, and binary outcomes (Tables 2 and 3 and Table S2). Pairs were treated as clusters and assigned random effect variables, to enable analysis of replicate BMFS sample results without bias. Pairs were defined as two‐phase and BMFS (individual or replicate) samples, collected within 5 min at a 1‐m radius of each other. Variables other than pairs and the target were considered precision variables and assigned fixed effects. Analyses that did not include random effect variables (i.e. pairs) were analysed by logistic regression. The logistic regression accounted for fixed effect variables and binary outcomes (Table 3 and Table S2). All variables were considered precision variables and assigned fixed effects, other than the target variable. For both the GLMM and logistic regression, all assayed samples were included for analysis of SL1, SL3, NPEV and any PV. For analyses on factors impacting SL2 detection, only samples prior to 18 July 2016 were considered, as SL2 was presumed absent from the environment 3 months after the bOPV switch (Huang *et al*. 2005). Results from these analyses included the OR of positive virus detection with an increase in the predictor of interest by one unit, while holding all other factors constant, the 95% CI for the OR, and the *P*‐value.

**Table 3:** Impact of contributing factors on positive PV detection in BMFS or two‐phase samples*

	OR	95% CI	*P*‐value	*n* ^†^	Adjustment factors
Filtration volume (BMFS): *GLMM*					
SL1	0·49	0·14, 1·76	0·272	217	Sample site, pair
SL2	2·37	0·48, 11·6	0·287	119	Sample site, pair, bOPV
SL3	0·54	0·21, 1·41	0·211	217	Sample site, pair
Filtration time (BMFS): *GLMM*					
SL1	1·10	0·39, 3·05	0·862	217	Sample site, pair
SL2	0·95	0·25, 3·68	0·945	119	Sample site, pair, bOPV
SL3	0·64	0·30, 1·36	0·244	217	Sample site, pair
Processing time (BMFS: CDC)^‡^: *GLMM*					
SL1	0·98	0·91, 1·05	0·550	121	Sample site, pair
SL2	1·13	0·96, 1·32	0·145	75	Sample site, pair, bOPV
SL3	0·97	0·90, 1·04	0·340	121	Sample site, pair
Processing time (BMFS: KEMRI)^§^: *Logistic regression*					
SL1	1·06	0·99, 1·13	0·100	100	Sample site
SL2	1·05	0·95, 1·17	0·320	44	Sample site, bOPV
SL3	1·15	1·03 1·29	0·015	100	Sample site
Sample transit time (BMFS: CDC)^‡^: *GLMM*					
SL1	0·89	0·71, 1·13	0·358	121	Sample site, min. temp., pair
SL2	2·15	0·21, 22·4	0·523	75	Sample site, min. temp., pair, bOPV
SL3	0·93	0·83, 1·04	0·210	121	Sample site, min. temp., pair
Sample transit minimum temperature (BMFS: CDC)^‡^: *Logistic regression*					
SL1	1·02	0·89, 1·15	0·816	75	Sample site, transit time
SL2	0·87	0·73, 1·03	0·110	39	Sample site, transit time, bOPV
SL3	0·97	0·86, 1·09	0·620	75	Sample site, transit time
Sample transit maximum temperature (BMFS: CDC)^‡^: *Logistic regression*					
SL1	0·98	0·91, 1·06	0·606	75	Sample site
SL2	1·05	0·84, 1·31	0·691	39	Sample site, bOPV
SL3	0·92	0·86, 0·99	0·027	75	Sample site
Duration of cold chain loss (BMFS: CDC)^‡^: *Logistic regression*					
SL1	0·91	0·80, 1·03	0·121	37	Sample site, max. temp.
SL2	0·80	0·31, 2·07	0·650	12	Sample site, max. temp., bOPV
SL3	1·05	0·95, 1·16	0·351	37	Sample site, max. temp.
Assay site (BMFS): *GLMM*					
SL1	1·41	0·69, 2·85	0·345	221	Sample site, pair
SL2	0·63	0·24, 1·67	0·358	119	Sample site, pair, bOPV
SL3	1·51	0·82, 2·79	0·183	221	Sample site, pair
Assay site (Two‐phase): *Logistic regression*					
SL1	3·78	0·83, 17·2	0·086	133	Sample site
SL2	1·21	0·31, 4·76	0·788	77	Sample site, bOPV
SL3	2·46	0·91, 6·66	0·076	133	Sample site

PV, poliovirus; BMFS, bag‐mediated filtration system; OR, odds ratio; 95% CI, 95% confidence interval boundaries; GLMM, generalized linear mixed model; SL1, Sabin‐like PV type 1; SL2, Sabin‐like PV type 2; SL3, Sabin‐like PV type 3.

* See Table S4 for a detailed explanation of the targets and adjustment factors included. Filtration volume (l, continuous); filtration time (hours, continuous); Processing time (days, continuous); Sample transit time (days, continuous); Sample transit minimum temperature (°C, continuous); Sample transit maximum temperature (°C, continuous); Duration of cold chain loss (hours, continuous); Assay site (binary); Sample site (categorical); Pair (cluster); and bOPV (binary).

^†^ See Table S2 for a detailed explanation of samples numbers included in analyses.

^‡^ Analysis included only samples that were assayed at CDC.

^§^ Analysis included only samples that were assayed at KEMRI.

The Pearson’s chi‐squared test was used to determine the likelihood that differences in virus detection before and after the bOPV switch were due to chance (Objective 3) (Zhou *et al*. 2018). The BMFS samples included are the same as used during the McNemar mid‐*P* analysis (Table S2). The OR of virus detection during tOPV use, compared to detection during bOPV use, was calculated.

## Results

### Comparison of PV and NPEV detection in BMFS and two‐phase samples

**Figure 2:**
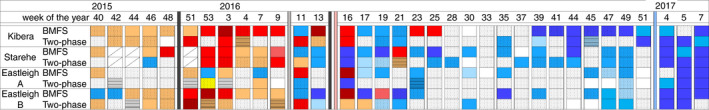
Poliovirus (PV) and non‐polio enterovirus (NPEV) detection in bag‐mediated filtration system (BMFS) and two‐phase samples. SL1 is Sabin‐like PV type 1. SL2 is Sabin‐like PV type 2. SL3 is Sabin‐like PV type 3. VDPV2 is vaccine‐derived PV type 2. NEV is non‐enterovirus. bOPV is bivalent oral polio vaccine. tOPV is trivalent oral polio vaccine (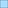 SL1; (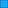 SL3; (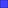 SL1 & SL3; (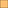 SL2; (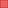 SL1 & SL2; (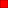 SL2 & SL3; (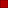 SL1, SL2 & SL3; (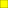 SL3 & VDPV2; (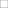 negative for PV; (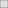 NPEV (alone or with PV); (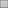 NPEV & NEV (alone or with PV); (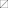 no data; (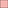 Vaccine switch to bOPV (removal of SL2); (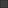 National Immunization Day (tOPV); (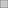 Sub‐National Immunization Day (tOPV); (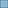 Sub‐National Immunization Day (bOPV).

Sabin‐like PV type 1, SL2 and/or SL3 was detected in a majority of BMFS (72·2%) and two‐phase (52·6%) samples (*n* = 133) (Fig. 2 and Table 2). WPV was not detected. Mixtures of PV serotypes were detected in BMFS (37·6%) and two‐phase (17·3%) samples (*n* = 133) (Fig. 2). There was no significant difference in SL1, SL2 or SL3 detection between replicate BMFS samples analysed at CDC compared to KEMRI (*P* = 0·839, 0·791 and 0·860, respectively, McNemar mid‐*P*; Table S1).

Sabin‐like PV type 1, SL2 and SL3 were detected at significantly higher frequency in BMFS than two‐phase samples, with a significantly greater OR calculated using the McNemar mid‐*P* test (2·38 (1·04, 5·43), *P* = 0·036; 3·17 (1·23, 7·93), *P* = 0·009; 3·90 (1·95, 7·81), *P* = 2 × 10^−5^, respectively) and GLMM (2·79 (1·32, 5·87), *P* = 0·007; 3·53 (1·40, 8·89), *P* = 0·007; 3·57 (2·10, 6·32), *P* = 1 × 10^−5^, respectively) (Table 1). NPEV was detected more frequently in two‐phase samples, with a significantly lower OR for BMFS using the McNemar mid‐*P* test (0·41 (0·22, 0·74), *P* = 0·002) and GLMM (0·50 (0·29, 0·85), *P* = 0·010) (Table 1).

### Factors impacting PV detection

The volume passed through each filter ranged between 1·4 and 4·0 l and averaged 2·7 ± 0·16 l (95% CI). The average concentration factor was 270‐fold, and average effective volume assayed was 815 ± 18 ml (95% CI). SL1, SL2 and SL3 detection were not statistically impacted by BMFS filtration volume (*P* = 0·272, 0·287 and 0·211, respectively) or filtration time (*P* = 0·862, 0·945, and 0·244, respectively) (Table 3). For BMFS samples analysed at KEMRI, an increased time from collection to obtaining primary concentrate resulted in significantly decreased odds of SL3 detection (*P* = 0·015). Assay location (KEMRI or CDC) did not statistically impact PV detection in BMFS or two‐phase samples (Fig. 1, Table 3).

Of 90 refrigerated BMFS filters shipped with temperature trackers, 48·9% lost cold chain during shipment (>8°C), with 4·4% exceeding 25°C. The average duration of cold chain loss was 31·4 ± 7·3 h (95% CI). BMFS filter shipping conditions did not impact SL1 or SL2 detection (*P *> 0·1, Table 3). Odds of SL3 detection significantly decreased with a higher maximum shipment temperature (0·92 (0·86, 1·09), *P* = 0·027; OR (CI)), though other shipping factors did not impact SL3 detection (*P *> 0·2).

### Presence of SL2 prior to tOPV withdrawal

During tOPV use (29 September 2015–18 April 2016), the most frequently detected PV was SL2, followed by SL3 and SL1 (Table 4). After the bOPV switch, SL2 was the least frequently detected PV. SL2 was detected more frequently before the bOPV switch (*P* < 1 × 10^−5^ for both BMFS and two‐phase samples), and no statistical difference in SL3 detection was observed (*P* = 0·134 and 0·084 for BMFS and two‐phase samples, respectively). SL1 was detected more frequently in BMFS and two‐phase samples (*P* = 0·003 and 0·014, respectively) after type 2 withdrawal.

**Table 4:** Effect of tOPV to bOPV switch on PV detection in BMFS and two‐phase samples

	tOPV use		bOPV use		Pearson’s chi‐squared test		
	Detection (%)	*n*	Detection (%)	*n*	χ^2^	*P*‐value	*n*
SL1							
BMFS	10·2	49*	33·3	84*	8·87	0·003	133*
Two‐phase	6·1	49	22·6	84	6·10	0·014	133
SL2							
BMFS	67·3	49*	8·3	84*	51·2	<0·001	133*
Two‐phase	46·9	49	4·8	84	34·0	<0·001	133
SL3							
BMFS	44·9	49*	58·3	84*	2·24	0·134	133*
Two‐phase	22·4	49	36·9	84	2·99	0·084	133

tOPV, trivalent oral polio vaccine; bOPV, bivalent oral polio vaccine; PV, poliovirus; BMFS, bag‐mediated filtration system; OR, odds ratio.

* BMFS samples included single (*n* = 30) and combined (*n* = 3) replicate samples analysed at CDC (29 September 2015 through 15 February 2016) and single samples analysed at KEMRI (*n* = 100; 16 February 2016 through 14 February 2017).

Before PV2 withdrawal, SL2 was frequently detected in BMFS (67·4%) and two‐phase (46·9%) samples (Fig. 2). After the final tOPV campaign (9–13 April 2016), SL2 was detected during 8 of 84 sampling events: 7 BMFS and 4 two‐phase. Final SL2 detection varied by site (22 April–23 June 2016).

## Discussion

The BMFS method detected SL1, SL2 and SL3 more frequently in environmental samples than the two‐phase method (*P* = 0·036, 0·009, and 2 × 10^−5^ (McNemar mid‐*P*) and *P = *0·007, 0·007, and 1 × 10^−5^ (GLMM), respectively). Processing variables (e.g. time, volume, transit conditions, location, etc.) did not impact SL1 and SL2 detection in BMFS samples, although processing time and maximum shipment temperature impacted SL3 detection. SL2 was detected less frequently after the bOPV switch, indicating a successful switch in Nairobi. This study demonstrated that BMFS can be an acceptable method for PV environmental surveillance and resulted in successful PV environmental surveillance in Nairobi.

Increased PV detection by BMFS may be due to the higher concentration factor (Zhou *et al*. 2018), with an average of 270‐fold for BMFS versus 50‐fold for two‐phase samples. However, the increase in PV detection is not directly proportional to the concentration factor and effective volume assayed, as the assay is nonquantitative.

When examining BMFS samples only, filtration volume (and consequently, concentration factor) did not impact SL1, SL2 or SL3 detection (*P* = 0·272, 0·287 and 0·211, respectively, Table 3). As 96·5% of BMFS samples filtered 2·0–3·5 l, additional data at lower and higher volumes may help examine the full effects of filtration volume on PV detection. Future research may determine an optimal volume at which PV would be detected in a majority of samples (with PV presence in the system).

The effective volume assayed in BMFS samples is due to concentration of the original volume (2·7 l) to the WHO algorithm target volume (10 ml), using both primary and secondary concentration. Secondary concentration is not used in the two‐phase method because it increases sample manipulation, and modifying this existing standard method would complicate harmonization around the global network, and the method already results in the current target volume for the WHO algorithm. Incorporating secondary concentration into the two‐phase method could be explored to increase the effective volume assayed if cell culture independent PV detection was used or if a greater concentration factor was desired.

Shipping and processing variables did not impact SL1 and SL2 detection in BMFS samples (*P *> 0·1), indicating viruses on BMFS filters can withstand shipment delays, long filtration times and cold chain loss. Odds of SL3 detection were reduced with an increased maximum shipment temperature (*P* = 0·027), suggesting SL3 sensitivity to temperature fluctuation. Preservative agent treatment on the filters helped maintain sample integrity when cold chain was lost during shipment (Fagnant *et al*. 2017a). Filtration time ranged from 89 to 240 min, indicating PV can be detected when the filtration system is placed in direct sunlight for extended times (Nairobi maximum temperatures average 24–28°C (Egondi *et al*. 2015)).

SL2 was the most frequently detected PV during tOPV use. While SL1, SL2 and SL3 are shed at similar rates, SL2 circulates more widely among unvaccinated individuals, thus increasing SL2 environmental prevalence (Troy *et al*. 2014; Ferreyra‐Reyes *et al*. 2017), and possibly contributing to frequent SL2 detection. After the switch to bOPV, SL2 detection decreased, suggesting its absence from the environment following OPV2 withdrawal in Nairobi. SL1 and SL3 were detected more frequently after the switch to bOPV and these results were statistically significant for SL1 (Table 4).

The study had several limitations (Zhou *et al*. 2018). Ten per cent of BMFS samples analysed at KEMRI were collected following the tOPV campaign from 9 to 13 April 2016 and experienced filter hold times of 19–26 days. As the tOPV would increase Sabin‐like PV shedding and subsequently increase environmental PV concentrations, these samples may have disproportionately impacted SL3 detection analyses. These showed improved odds of SL3 detection, with increased time from collection to primary concentration when BMFS samples were analysed at KEMRI (OR = 1·15; Table 3). While BMFS and two‐phase samples were collected sequentially within a 1‐m radius, they were not processed from the same homogenous source, thus natural virus distribution is reflected in the results. Additionally, as the Poliovirus Isolation Algorithm was utilized for analysis and is designed for PV detection, PV presence may have impacted NPEV reporting, which is used as a site and sample control. As the BMFS detected PV more frequently than two‐phase during this study, it is difficult to compare the rate of NPEV reporting between BMFS and two‐phase samples due to potential masking of NPEVs in a PV background. Finally, use of MS2 as an internal process control yields inconsistent recoveries, potentially due to disaggregation, integrity of the MS2, challenges with the double agar layer assay or other issues. Future work should examine the use of alternative process controls, be it seeded or indigenous organisms such as adenovirus, pepper mild mottle virus, other bacteriophages, or direct detection of NPEV.

The BMFS resulted in frequent PV detection. Generally, filtration, processing and filter shipping variables did not impact PV detection in BMFS samples, indicating that BMFS retains sample integrity even under nonoptimal conditions. SL2 was detected less frequently after the bOPV switch, indicating that the gradual decrease of SL2 is commensurate with OPV2 withdrawal. The BMFS offers comparable or improved PV detection under the conditions of this study, relative to the two‐phase method. Future BMFS work should explore its ability to detect additional targets, including other viruses, bacteria, parasites and antimicrobial resistance genes.

## Conflict of Interest

No conflict of interest declared.

## Author contributions

All authors contributed to reviewing and editing the manuscript. Christine Fagnant‐Sperati, Yuqi Ren and Nicolette Zhou contributed to data curation, formal data analysis, methodology design, resources, data validation, data visualization and writing of the original manuscript draft. Evans Komen and Stacey Miles contributed to data curation, investigation and resources. Benlick Mwangi, Joanne Hassan, Agnes Chepkurui, Peter Matsapola, Fhatuwanti Ngwana and Silvia Penaranda contributed to the investigation and resources. Rosemary Nzunza and Marianne Wolfaardt contributed to the investigation, resources and supervision. James Nyangao and Angela Coulliette‐Salmond contributed to the investigation, project administration, resources, and supervision. Walda van Zyl contributed to data curation, investigation, project administration, resources and supervision. Everardo Vega contributed to the investigation, project administration, resources, supervision, data visualization and writing of the original manuscript draft. Jeffry Shirai and Nicola Beck contributed to the methodology design, project administration and resources. Alexandra Kossik contributed to the methodology design and resources. David Boyle contributed to the conceptualization, funding acquisition, methodology design and supervision. Cara Burns, Maureen Taylor and Peter Borus contributed to the methodology design and supervision. John Scott Meschke contributed to the conceptualization, data curation, funding acquisition, methodology design and supervision.

## Supporting information


**Appendix S1**. Nairobi environmental surveillance sites.
**Appendix S2**. Statistical methods.
**Appendix S3**. Replicate BMFS samples.
**Appendix S4**. Samples included in statistical analyses.
**Appendix S5**. NPEV detection in BMFS and two‐phase samples.
