# Supplementary material for: Validation of the bag‐mediated filtration system for environmental surveillance of poliovirus in Nairobi, Kenya
Source: J Appl Microbiol. 2020 Aug 14;130(3):971–81. doi: 10.1111/jam.14807 (PMC7854911; doi:10.1111/jam.14807)
Supplement: Supplementary file 1 — Appendix S1. Nairobi environmental surveillance sites. Appendix S2. Statistical methods. Appendix S3. Replicate BMFS samples. Appendix S4. Samples included in statistical analyses. Appendix S5. NPEV detection in BMFS and two‐phase samples. [file JAM-130-971-s001.docx]

SUPPORTING INFORMATION

*Validation of the bag-mediated filtration system for environmental surveillance of poliovirus in Nairobi, Kenya*

**Authors**

Christine Susan Fagnant-Sperati^1^, Yuqi Ren^2^, Nicolette Angela Zhou^1^, Evans Komen^3^, Benlick Mwangi^3^, Joanne Hassan^3^, Agnes Chepkurui^3^, Rosemary Nzunza^3^, James Nyangao^3^, Walda Brenda van Zyl^4^, Marianne Wolfaardt^4^, Peter Nameng Matsapola^4^, Fhatuwani Britton Ngwana^4^, Stacey Jeffries-Miles^5^, Angela Coulliette-Salmond^6^, Silvia Peñaranda^6^, Everardo Vega^6^, Jeffry Hiroshi Shirai^1^, Alexandra L. Kossik^1^, Nicola Koren Beck^1^, David S. Boyle^7^, Cara C. Burns^6^, Maureen Beatrice Taylor^4^, Peter Borus^3^, John Scott Meschke^1^*

**Author Addresses**

^1^ Department of Environmental and Occupational Health Sciences, University of Washington

^2^ Department of Biostatistics, University of Washington

^3^ Centre for Viral Research, Kenya Medical Research Institute

^4^ Department of Medical Virology, University of Pretoria

^5^ Cherokee Nation Assurance a contracting agency to the Division of Viral Diseases, Centers for Disease Control and Prevention

^6^ Division of Viral Diseases, Centers for Disease Control and Prevention

^7^ PATH; 2201 Westlake Ave, Suite 200, Seattle, WA 98121, USA

**Corresponding Author**

*corresponding author: email: [jmeschke@uw.edu](mailto:jmeschke@uw.edu); phone: +1-206-221-5470; postal: Department of Environmental and Occupational Health Sciences, University of Washington; 4225 Roosevelt Way NE, Suite 100, Seattle, WA 98125, USA

*Submitted to Journal of Applied Microbiology*

**Table of Contents**

[*Nairobi Environmental Surveillance Sites* 4](#_Toc35245516)

[*Statistical Methods* 5](#_Toc35245517)

[*Replicate BMFS Samples* 8](#_Toc35245518)

[**Table S1.** Comparison of PV detection in replicate BMFS samples analyzed at KEMRI or CDC 8](#_Toc35245519)

[*Samples included in statistical analyses* 9](#_Toc35245520)

[**Table S2.** BMFS and two-phase samples included in statistical analyses 9](#_Toc35245521)

[*NPEV detection in BMFS and two-phase samples* 11](#_Toc35245522)

[**Table S3.** Effect of positive NPEV detection on PV detection in BMFS and two-phase samples 11](#_Toc35245523)

# *Nairobi Environmental Surveillance Sites*

Sites included a sewer conveyance line accessed via its outlet in the Mathare informal settlement (Starehe), two sewer conveyance lines accessed via manhole in the Eastleigh neighborhood (Eastleigh A and B), and an open channel bordering the Kibera informal settlement (Kibera). Wastewater infrastructure maps and risk of wild poliovirus occurrence informed site selection.

# *Statistical Methods*

The McNemar mid-*p* test was used to determine the significance of the difference between paired, sequentially collected samples (Eq S1).

$$\text{mid-}\text{p}\text{-value}=\text{2}\left( \sum_{x_{12}=0}^{min\left( n_{12},n_{21} \right)} \left( \begin{matrix} n \\ x_{12} \end{matrix} \right)\left( \frac{1}{2} \right)^{n} \right)-\left( \begin{matrix} n \\ n_{12} \end{matrix} \right)\left( \frac{1}{2} \right)^{n} (Eq S1)$$

where *n_12_* is the number of matched samples discordant in favor of the bag-mediated filtration system (BMFS), *n_21_* is the number of matched samples discordant in favor of two-phase, *n* is the sum of the discordance (*n_12_* + *n_21_*), and *x_12_* is 0, 1, …, *min(n_12_,n_21_)*. Results were considered significant with a mid-*p*-value < 0.05.

The odds ratio (OR) and confidence interval (CI) on the OR were then calculated (Eq S2, Eq S3).

$$OR=\frac{n_{12}}{n_{21}} (Eq S2)$$

$$95\% CI=e^{lnOR\pm z\times\sqrt{\frac{1}{n_{12}}+\frac{1}{n_{21}}}} (Eq S3)$$

where z is 1.96 for 95% confidence.

The Pearson’s chi-squared test determined if the likelihood that the differences in virus detection before and after the switch to the bivalent oral polio vaccine were due to chance (Eq S4).

$$\chi^{2}=\frac{{n\left( ad-bc \right)}^{2}}{(a+b)(c+d)(a+c)(b+d)} (Eq S4)$$

where *a* is the number of samples positive during trivalent oral polio vaccine (tOPV) use, *b* is the number of samples negative during tOPV use, *c* is the number of samples positive during bivalent oral polio vaccine (bOPV) use, *d* is the number of samples negative during bOPV use, and *n* is the total number of samples.

The generalized linear mixed model (GLMM) was performed, to include random and non-random effect variables (Eq S5).

$$\log(odds(Y_{ij}))=\beta_{0}+\beta_{1}X_{ij}+\beta_{n}W_{ij}+\gamma_{i}Z_{i}+\varepsilon_{ij} (Eq S5)$$

where *i* represents water samples; *j* represents observations of each water sample; β_0_, β_1_, and β_n_ are fixed effects with specific values; γ is a random effect with normal distribution (0, τ_2_); Y is the outcome; X is the predictor of interest; W is a predictor with fixed effects; Z is a predictor with random effects; and ε_ij_ is the residual term with normal distribution (0, σ^2^).

As outcome variables were binary, the model estimated the probability that the outcome was 1. The results were transformed to a natural log of the odds that the outcome would result in the variable of interest. The odds were calculated (Eq S6) and the final result was interpreted as an odds ratio (Eq S7).

$$O\left( n \right)=\frac{P_{n}}{1-P_{n}} (Eq S6)$$

where P_n_ is the probability an event happens.

$$OR=\frac{\frac{P_{1}}{1-P_{1}}}{\frac{P_{2}}{1-P_{2}}} (Eq S7)$$

where P_1_ is the probability the outcome of interest occurs when the predictor of interest is positive and P_2_ is the probability the outcome of interest occurs when the predictor of interest is negative.

The logistic regression was performed, to include non-random effect variables (Eq S5).

$$\log(odds(Y_{ij}))=\beta_{0}+\beta_{1}X_{i}+\beta_{n}W_{ni} (Eq S8)$$

where *i* represents water samples; β_0_, β_1_, and β_n_ are fixed effects with specific values; Y is the outcome; X is the predictor of interest; and W_n_ is a predictor with fixed effects.

The outcome variables were binary, and so the model estimated the probability that the outcome was 1. The results were transformed to a natural log of the odds that the outcome would result in the variable of interest. The odds were calculated (Eq S6) and the final result was interpreted as an odds ratio (Eq S7).

# *Replicate BMFS Samples*

## **Table S1.** Comparison of PV detection in replicate BMFS samples analyzed at KEMRI or CDC

| SL1 | | KEMRI | |  | SL2 | | KEMRI | |  | SL3 | | KEMRI | |
| --- | --- | --- | --- | --- | --- | --- | --- | --- | --- | --- | --- | --- | --- |
|  |  | + | - |  |  |  | + | - |  |  |  | + | - |
| CDC | + | 14 | 12 |  | CDC | + | 8 | 6 |  | CDC | + | 32 | 15 |
|  | - | 11 | 48 |  |  | - | 7 | 64 |  |  | - | 16 | 22 |
| OR (CI) | | 0.92 (0.40, 2.08) | |  | OR (CI) | | 1.17 (0.39, 3.47) | |  | OR (CI) | | 1.07 (0.53, 2.16) | |
| *p*-value | | 0.839 | |  | *p*-value | | 0.791 | |  | *p*-value | | 0.860 | |

KEMRI, Kenya Medical Research Institute; CDC, Centers for Disease Control and Prevention; OR, Odds ratio; CI, 95% confidence intervals.

# *Samples included in statistical analyses*

## **Table S2.** BMFS and two-phase samples included in statistical analyses

| Predictor of interest | BMFS | | | | | | |  | Two-phase | | | | | | |
| --- | --- | --- | --- | --- | --- | --- | --- | --- | --- | --- | --- | --- | --- | --- | --- |
|  | Analysis method | SL1, SL3 | | | SL2 | | |  | Analysis method | SL1, SL3 | | | SL2 | | |
|  |  | *n*^*^ | Per.^†^ | Assay site (*n*) | *n* | Per. | Assay site (*n*) |  |  | *n* | Per. | Assay site (*n*) | *n* | Per. | Assay site (*n*) |
| Sample method | McNemar mid-*p* test | 133 | 1  2 | CDC (33) KEMRI (100) | 133 | 1  2 | CDC (33) KEMRI (100) |  | McNemar mid-*p* test | 133 | 1  2 | CDC (33) KEMRI (100) | 133 | 1  2 | CDC (33) KEMRI (100) |
|  | GLMM | 221 | 1  2  2 | CDC (36) CDC (85) KEMRI (100) | 221 | 1  2  2 | CDC (36) CDC (85) KEMRI (100) |  | GLMM | 133 | 1  2 | CDC (33) KEMRI (100) | 133 | 1  2 | CDC (33) KEMRI (100) |
| Filtration volume | GLMM | 217 | 1  2  2 | CDC (36) CDC (85) KEMRI (96) | 119 | 1  2b | CDC (36) CDC (42) KEMRI (44) |  | n/a |  |  | n/a |  |  | n/a |
| Filtration time | GLMM | 217 | 1  2  2 | CDC (36) CDC (85) KEMRI (96) | 119 | 1  2b  2b | CDC (36) CDC (42) KEMRI (44) |  | n/a |  |  | n/a |  |  | n/a |
| Process. time | GLMM (CDC) | 121 | 1  2 | CDC (36) CDC (85) | 75 | 1  2b | CDC (36) CDC (39) |  | n/a |  |  | n/a |  |  | n/a |
|  | Logistic regression (KEMRI) | 100 | 2 | KEMRI (100) | 44 | 2b | KEMRI (44) |  | n/a |  |  | n/a |  |  | n/a |
| Sample transit time | GLMM | 121 | 1  2 | CDC (36) CDC (85) | 75 | 1  2b | CDC (36) CDC (39) |  | n/a |  |  | n/a |  |  | n/a |
| Min. temp. | Logistic regression | 75 | 1  2 | CDC (7) CDC (68) | 39 | 1  2b | CDC (7) CDC (32) |  | n/a |  |  | n/a |  |  | n/a |
| Max. temp. | Logistic regression | 75 | 1  2 | CDC (7) CDC (68) | 39 | 1  2b | CDC (7) CDC (32) |  | n/a |  |  | n/a |  |  | n/a |
| Duration of cold chain loss | Logistic regression | 37 | 1  2 | CDC (4) CDC (29) | 12 | 1  2b | CDC (4) CDC (8) |  | n/a |  |  | n/a |  |  | n/a |
| Assay site | GLMM | 221 | 1  2  2 | CDC (36) CDC (85) KEMRI (100) | 119 | 1  2b  2b | CDC (36) CDC (42) KEMRI (44) |  | Logistic regression | 133 | 1  2 | CDC (33) KEMRI (100) | 77 | 1  2b | CDC (33) KEMRI (44) |
| bOPV switch | Pearson's χ² test | 133 | 1  2 | CDC (33) KEMRI (100) | 133 | 1  2 | CDC (33) KEMRI (100) |  | Pearson's χ² test | 133 | 1  2 | CDC (33) KEMRI (100) | 133 | 1  2 | CDC (33) KEMRI (100) |

*n*: number of samples included in statistical analysis; Per.: period of the project; Sample method: concentrated by BMFS or two-phase method; Filtration volume: volume filtered in BMFS samples (L); Filtration time: time required for sample filtration (minutes); Process. time: processing time between BMFS sample collection to elution (days); Sample transit time: time BMFS filter was in transit from KEMRI to UP (days); Min. temp.: minimum temperature reached by BMFS filter during shipment from KEMRI to UP (°C); Max. temp.: maximum temperature reached by BMFS filter during shipment from KEMRI to UP (°C); Duration of cold chain loss: amount of time BMFS filter experienced temperatures greater than 8°C during shipment from KEMRI to UP (hours); Assay site: sample assayed at KEMRI or CDC; bOPV switch: sample collection before or after switch PV2 withdrawal from the oral polio vaccine; GLMM, generalized linear mixed model.

^*^ Samples with relevant chain-of-custody forms were included in analyses

^†^ Period 1: 29 September 2015 to 15 February 2016; Period 2: 16 February 2016 to 14 February 2016; Period 2b: 16 February 2016 to 18 July 2016

# *NPEV detection in BMFS and two-phase samples*

## **Table S3.** Effect of positive NPEV detection on PV detection in BMFS and two-phase samples

|  | OR | 95% CI | *p*-value | *n*^*^ | Adjustment factors |
| --- | --- | --- | --- | --- | --- |
| BMFS: *GLMM* | | | | | |
| SL1 | 0.13 | 0.04, 0.40 | <0.001 | 221 | Sample site, season, pair |
| SL2 | 0.15 | 0.05, 0.43 | <0.001 | 119 | Sample site, season, pair, bOPV |
| SL3 | 0.12 | 0.05, 0.31 | <0.001 | 221 | Sample site, season, pair |
| Two-phase: *Logistic regression* | | | | | |
| SL1 | 0.12 | 0.04, 0.35 | <0.001 | 133 | Sample site, season |
| SL2 | 0.29 | 0.07, 1.22 | 0.092 | 77 | Sample site, season, bOPV |
| SL3 | 0.09 | 0.04, 0.22 | <0.001 | 133 | Sample site, season |

NPEV, non-polio enterovirus; PV Negative, no poliovirus detected in sample; OR, odds ratio; CI, 95% confidence intervals; *p*-value, calculated by the generalized linear mixed model (BMFS samples) or logistic regression (two-phase samples).

^*^ All BMFS samples were included in analysis.
